# Dual Process Theory of Thought and Default Mode Network: A Possible Neural Foundation of Fast Thinking

**DOI:** 10.3389/fpsyg.2018.01237

**Published:** 2018-07-17

**Authors:** Giorgio Gronchi, Fabio Giovannelli

**Affiliations:** Section of Psychology, Department of Neuroscience, Psychology, Drug Research and Child's Health, University of Florence, Florence, Italy

**Keywords:** dual process theory of thought, default mode network (DMN), neural basis of cognition, decision making, automatic behavior

## Introduction

The notion of default mode network (DMN) and the dual process theory of thought, topics within different cognitive neuroscience and psychology subfields, have attracted considerable attention and been extensively studied in the past decade. The former originated from experimental evidence on the brain function obtained when an individual is not involved in a specific task, and recent research suggests that the DMN plays a role in mental and neurological disorders (Buckner et al., 2008). A distinction is made according to the psychology of thinking in the dual process theory of thought between fast, effortless associative processes and slow, deliberative ones (Kahneman, 2011). This theory has been exploited both theoretically, to better understand human thought, and in many applications of behavior modification (Thaler and Sunstein, 2008).

This paper proposes that an amalgamation of the aspects of these two topics could be of mutual benefit to scientists within the respective fields. The discovery of the DMN has stimulated several hypotheses regarding the neural basis of the self and the theory of the mind. However, with few exceptions, these hypotheses lack reference to current research on thought processes like reasoning and decision-making. A role of the DMN in the organization and expression of preplanned, reflexive behaviors characteristics of fast thinking has been mentioned by Raichle (2015). Moreover, a link between fast and slow processes and the activity of neural circuits including the DMN, has been proposed in the framework of the Predictive And Reactive Control Systems (PARCS) theory (Tops et al., 2014). More recently, the contribution of the DMN to automated processing has been also suggested (Vatansever et al., 2017). In contrast, the dual process theory of thought is the most shared explanation of how thoughts arise but does not adequately address the neural basis of thought, although an attempt has been made to determine the relationship between ego depletion and biological parameters (Elkins-Brown et al., 2016). Thus, in our opinion the DMN may provide a neural foundation for the associative, fast, and effortless form of thinking elucidated by the dual process theory.

## The dual process theory of thought

Since the earliest days of philosophical enquiries into the mind, many researchers have entertained the idea that two different systems of thought co-exist; a quick, automatic, associative, and affective-based form of reasoning and a slow, thoughtful, deliberative process (Sloman, 1996, 2014; Epstein and Pacini, 1999; Lieberman, 2003; Stanovich, 2004; Kahneman and Frederick, 2005; Evans, 2006). Today, the psychology of thinking calls this idea “the dual process theory of thought” (Evans, 2003, 2008; Osman, 2004; Evans and Stanovich, 2013), which encompasses a variety of theories with different approaches to the processes involved in thought. The differences are reflected by the terminology. For example, the two co-existing processes have variously been dubbed System 1 vs. System 2 (Stanovich, 1999, 2004; Kahneman and Frederick, 2005), intuition vs. deliberation (Sloman, 2014), associative vs. rule-based thinking (Sloman, 1996), and fast vs. slow thinking (Kahneman, 2011). Broadly speaking, fast thinking is quick, effortless, associative and experience-based, and, according to some authors (e.g., Epstein, 1994; Sloman, 2014) is organized in a positive-feedback loop involving affective processes. By contrast, slow thinking requires effort and the use of cognitive resources, and is based on symbolic and abstract rule manipulation.

According to Evans (2007) there are two ways in which the two processes might interact. Parallel models (Denes-Raj and Epstein, 1994; Sloman, 1996) suggest that fast and slow thinking occur simultaneously (and thus there is a continuous monitoring and feelings of conflict). In contrast, Default-Interventionist (DI) models (De Neys and Glumicic, 2008; Evans and Stanovich, 2013) claim that fast thinking generates intuitive default responses in which subsequent slow thinking processing may or may not serially intervene (provided that adequate resources are available). However, recent and deeper analysis of the two models' assumptions sustains an “hybrid two-stage model” (De Neys and Glumicic, 2008; Thompson, 2013; Newell et al., 2015) in which a “shallow analytic monitoring process” is always active to detect potential conflicts between the two systems, and an “optional deeper processing stage” is activated once an actual conflict between fast and slow thinking is found. Sloman (2014) points out that the distinction between the two cannot be explained as a simple discrepancy between conscious and unconscious processes, or between rational and irrational processes. Indeed, it is possible through introspection to be conscious of either form of thinking. The difference is that one can have conscious awareness of the various processing steps involving in slow thinking, but only the output of the fast thinking reasoning process. In addition, either thinking modality can lead to rational or irrational conclusions being drawn. In particular, with fast thinking, sophisticated causal reasoning conclusions can be formed, based on normative principles (Sloman, 2014).

Fast thinking involves conditions of “cognitive ease” (Kahneman, 1973, 2011), whereby an individual tends to spontaneously think, choose, and act according to domain-specific and associative principles in situations that are easy to understand and process. Thus, inhibition by slow thinking is unnecessary. Slow thinking requires mental effort as measured by biological indices, such as pupil dilation (Kahneman and Beatty, 1966; Kahneman, 2011). Typically, mental effort is required for tasks requiring attention. Under these conditions, the individual is subject to a phenomenon called “ego-depletion:” When forced to do something, he or she has fewer cognitive resources available to activate slow thinking and thus it is less able to exert self-control (Baumeister et al., 1998; Muraven et al., 1998). However, a recent meta-analysis (Carter et al., 2015) and a multilab replication study (Hagger and Chatzisarantis, 2016) challenged the idea that self-control depends on cognitive resources limitations. The current debate seems to support a domain-specific conception of the ego depletion effect that is strongly affected by individual differences (Dang et al., 2013; Dang, 2016).

## The default mode network

Twenty years ago, the convergence of much experimental evidence lent support to the premise that the “resting state” (i.e., the baseline task state used as the control in functional magnetic resonance imaging and positron emission tomography studies) is spontaneously active during periods of “passivity” (Biswal et al., 1995; Biswal, 2012; Buckner, 2012; Snyder and Raichle, 2012). The idea of DMN has its roots in the neuroimaging research revealing task-induced activity decreases from a resting state in a set of brain regions that were characterized for the first time by Shulman et al.'s (1997) in a meta-analytic study. These observations, along with findings reporting high metabolic activity in these regions at rest (Raichle et al., 2001), led to the widely acknowledged introduction of the DMN concept and constituted the first clear evidence of the existence of a cohesive default mode in the brain (Raichle et al., 2001). Subsequent studies demonstrated that the main nodes of the DMN are functionally and structurally connected (Greicius et al., 2003, 2009). The brain regions involved in the DMN include the medial prefrontal cortex, the posterior cingulate cortex, the inferior parietal lobule, the lateral temporal cortex, the dorsal medial prefrontal cortex, and the hippocampal formation (Buckner et al., 2008). The DMN is characterized by lower activity levels during goal-directed cognition or when a person is engaged in a particular task requiring externally directed attention, and higher activity levels when awake and involved in mental processes requiring low attentional demands. Given the association between the DMN and states in which thought is focused on internal channels, the DMN is generally considered the neural basis of spontaneous cognition (Buckner et al., 2008) and is responsible for everything that occurs while thinking using internal representations -including “stream of consciousness” (daydreaming and memory recall, particularly autobiographical memory) (James, 1890), envisioning the future, monitoring the environment, and thinking about others' intentions.

Spontaneous cognition is receiving increased attention, motivating researchers to renew previous routes of investigation and introduce novel methods and experimental paradigms (Smallwood and Schooler, 2006). An example of such an experimental paradigm involves the idea of stimulus-independent thoughts (SITs), which are defined (Buckner et al., 2008, p. 15) as “thoughts about something other than events originating from the environment” that are covert and not directed toward performance of the task at hand According to Buckner et al. (2008), the most widely used method of evaluating SITs involves periodically probing trained participants to indicate whether or not they are experiencing an SIT. SITs have been researched since the 1960s, albeit under a different classification (Antrobus, 1968). However, interest in this topic has increased, which, after the dissemination of the DMN concept, led to exploration of the relationship between neural activity and SITs (McKiernan et al., 2003, 2006) and individual differences (Mason et al., 2007).

Two main hypotheses have been proposed to explain DMN function; the internal mentation hypothesis and the sentinel hypothesis (Buckner et al., 2008). According to the former, the DMN plays a role in self-referential processes, i.e., internal mentation about social and emotional content (Mitchell et al., 2006), mental simulation, theory of mind-related considerations, and moral decision-making concerning personal moral dilemmas (Greene et al., 2001). By contrast, the sentinel hypothesis claims that the DMN helps to monitor the external environment (i.e., the direct opposite of focused attention toward a specific task), fulfilling “the continuous provision of resources for spontaneous, broad, and exogenously driven information gathering” (Hahn et al., 2007, p. 10). Very recently, the role of the DMN was highlighted in automatic behavior (the rapid selection of a response to a specific and predictable context) (Vatansever et al., 2017), as opposed to controlled decision-making, suggesting that the DMN plays a role in the autopilot mode of brain functioning.

## Discussion and conclusion

A few recent papers have begun to connect the two distinct subfields sketched above, from both a theoretical (Raichle, 2015) and empirical perspective (Vatansever et al., 2017). Indeed, several potential similarities exist between the dual-process theory of thought and the DMN, as illustrated in this paper. For example, according to the dual process theory of thought, fast thinking reflects conditions of cognitive ease (Kahneman, 1973, 2011), which is congruent with the premise of “spontaneous cognition” cited in the DMN literature. Similarly, the cognitive resources available to monitor the environment (the sentinel hypothesis) parallel those available in a state of cognitive ease and are reduced in conditions under which the ego is depleted (dual process theory). Beyond these general similarities, recent findings by Vatansever et al. (2017) on the possible role of the DMN as a neural basis of an autopilot system for human decision-making reflect the fast thinking-based decision process.

PARCS theory may play a seminal role in integrating these two subfields since it integrates a dual-process view similar to that of dual process theory of thought (Carver et al., 2008) with a cognitive neuroscience framework (Tops et al., 2010, 2014, 2015, 2017) that already comprises the DMN for fast processes. Moreover, PARCS theory is also able to explain the specific contexts in which the ego depletion effect holds in a way that is compatible with dual process theories (Tops, 2017). However, there is also evidence of incongruent features and a lack of correlation between the two theories—for example, the absence of a clear link between autobiographical memory (or envisioning the future) and fast thinking.

Given that the few works have been published on the subject and the potential similarities between DMN and dual process theories of thought, we advocate a deeper and more systematic investigation of the several parallelisms (and limitations) between the two ideas. DMN function could be better identified using the well-structured corpus of the dual-process theory of thought, while the DMN could constitute a potential neural basis for use in the dual process theory, thus creating a bridge between the psychology of thinking and neuroscience.

## Author contributions

GG and FG equally contributed to all phases of the development of the manuscript including conception, literature review, and writing.

### Conflict of interest statement

The authors declare that the research was conducted in the absence of any commercial or financial relationships that could be construed as a potential conflict of interest.
